# Neuronal and Cardiovascular Potassium Channels as Therapeutic Drug Targets: Promise and Pitfalls

**DOI:** 10.1177/1087057115601677

**Published:** 2015-10

**Authors:** Edward S. A. Humphries, Caroline Dart

**Affiliations:** 1 Institute of Integrative Biology, University of Liverpool, UK

**Keywords:** K^+^ channels, ion channels, channelopathies, cardiac, neuronal, vascular, drug development, review

## Abstract

Potassium (K^+^) channels, with their diversity, often tissue-defined distribution, and critical role in controlling cellular excitability, have long held promise of being important drug targets for the treatment of dysrhythmias in the heart and abnormal neuronal activity within the brain. With the exception of drugs that target one particular class, ATP-sensitive K^+^ (K_ATP_) channels, very few selective K^+^ channel activators or inhibitors are currently licensed for clinical use in cardiovascular and neurological disease. Here we review what a range of human genetic disorders have told us about the role of specific K^+^ channel subunits, explore the potential of activators and inhibitors of specific channel populations as a therapeutic strategy, and discuss possible reasons for the difficulty in designing clinically relevant K^+^ channel modulators.

## Potassium Ion Channels

**Figure 1.**
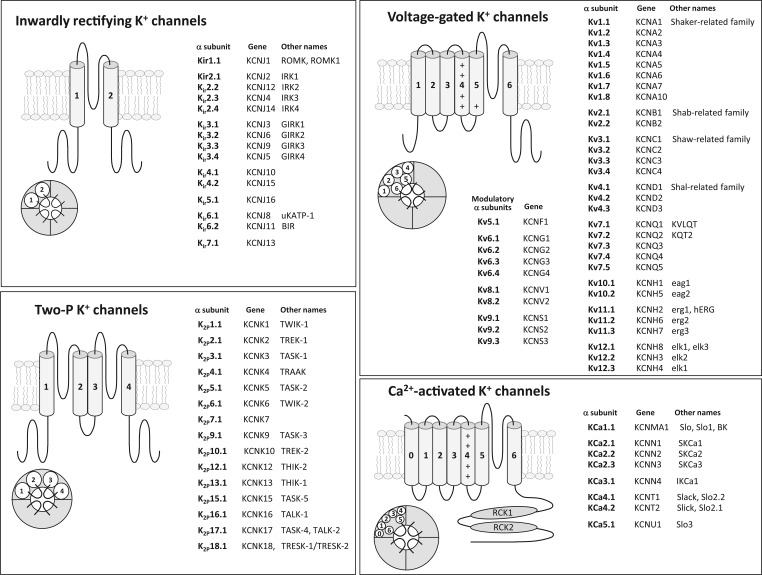
Schematic structure of the four main K^+^ channel classes as described by the International Union of Pharmacology.^1–4^ RCK, regulator of conductance for K^+^.

Potassium (K^+^) channels are a large family of integral membrane proteins that form aqueous pores in cell membranes through which K^+^ can flow. They are unique among ion channels in that they are found in virtually all types of cells in all organisms where they perform a range of biological functions. In the human genome, K^+^ channels are by far the largest and most diverse of the ion channel families, with almost 80 different genes encoding the principal pore-forming subunits (**Fig. 1**).^1–4^ In most cells, they play an essential role in maintaining and stabilizing the resting membrane potential. The opening of K^+^ channels, which occurs in response to a range of different signals, leads almost universally to the efflux of K^+^ from the cell, causing the membrane potential to become more negative. In nerve and muscle cells, their ability to repolarize or hyperpolarize the membrane helps them control action potential frequency and duration, while other functions include regulation of neurotransmitter release and hormone secretion, potassium homeostasis, epithelial electrolyte transport, cell proliferation, apoptosis, and tumor progression.^5–8^

Disruption of genes encoding K^+^ channel subunits and subsequent loss or gain of channel function is linked to a range of human diseases, including hyperinsulinemia, disturbances of the heart rhythm, and some types of epilepsy.^5,9,10^ K^+^ channels can also be subject to pathological inhibition by autoantibodies, leading to diseases such as acquired neuromyotonia^11^ and certain forms of epilepsy and encephalitis.^12–15^ These disorders have often helped to clarify the roles of particular channel populations within complex physiological systems and raise the possibility that activation or inhibition of selective K^+^ currents within cells could be a viable therapy. Indeed, K^+^ channel modulators are common medicines in certain diseases^16^; for example, in the treatment of diabetes, the oral antihyperglycemics such as glibenclamide, nateglinide, and glipizide all inhibit adenosine triphosphate (ATP)–sensitive (K_ATP_) channels. Most of the type III antiarrhythmics, including amiodarone, increase the cardiac refractory period by blocking several different types of K^+^ channel.^17^ In both epilepsy and hypertension, there are examples of drugs that target K^+^ channels.^16^ However, considering the scope for clinical impact, their membrane localization, diversity, and often defined tissue distributions, K^+^ channels remain underexploited as a target in drug discovery. This may be due to a number of factors. The sheer diversity of K^+^ channel subunits and their ability to form heteromultimeric complexes with different pore-forming subunits and accessory proteins means that the precise composition of functional channels within a particular tissue in vivo is often ill-defined. This makes predicting the functional outcome of channel loss or activation difficult and leads to unpredictable side effects even for specific activators/inhibitors. Drug screening programs also seem to find it difficult to identify selective channel activators. The direct measurement of ion channel activity by manual patch clamping is the best approach for assaying ion channel function but is time-consuming and unsuited to high-throughput screening (HTS). HTS techniques such as ligand binding or ion ﬂux assays, on the other hand, lack the complexity or resolution to detect subtle shifts in channel-gating kinetics that functionally may have profound effects on channel activity. The introduction of screening methods based around fluorometric dyes, which measure changes in ion concentration or cell membrane potential, coupled to fluorescent plate readers with in-built electrical ﬁeld stimulators, has improved temporal resolution but still represents an indirect measurement of channel activity.^18^ More recent advances in automated electrophysiology using planar-array patch-clamp technology circumvent many of the problems of functional resolution and have the potential to screen large compound libraries,^19^ and yet selective compounds remain elusive. One reason for this may be that channel modifiers often need to bind to relatively inaccessible sites within the channel pore or in clefts on or near regulatory domains. These sites may be relatively unforgiving to small structural changes within compounds, making chemical optimization of lead structures difficult. In this context, it is interesting to note that the K^+^ channel family that has the highest proportion of clinically used activators/inhibitors is the ATP-sensitive K^+^ (K_ATP_) channel family where modifiers appear to work by interacting at exposed peripheral sites on accessory subunits.^20^

Given their newly discovered roles in regulating cell proliferation and promoting tumor progression, drugs acting on K^+^ channels are likely to be of increasing clinical relevance.^7,21–23^ Here we review what disease-causing genetic disruption of specific K^+^ channel subunits in humans has told us about the functional role of K^+^ channels, explore the potential of selective activators and inhibitors as a therapeutic strategy, and further discuss possible reasons for the difficulty in designing clinically relevant K^+^ channel modulators. Due to the breadth of the field, we have limited our scope to K^+^ channels within cells of the human nervous and cardiovascular systems. A number of excellent recent reviews exist on disease-causing mutations in K^+^ channels within tissues such as the pancreas and nonvascular muscle and the role of drugs that target these channels. We will direct the reader to these, and others, throughout.

## K^+^ Channel Diversity

Structurally, K^+^ channels form from the association of (usually) four pore-forming α subunits often in association with modulatory accessory subunits. They can be grouped into the following four major classes^1–4^ (**Fig. 1**):

1. **Inwardly rectifying K^+^ (**K_ir_ **) channels**. In terms of structure, the K_ir_ family is the simplest K^+^ channel, with each subunit formed of just two transmembrane domains separated by a pore-forming region. These subunits form tetramers (four subunits) to produce functional K_ir_ channels.^24^ The family consists of the strong inwardly rectifying potassium channels (K_ir_2.x), the G protein–activated inwardly rectifying potassium channels (K_ir_3.x), and ATP-sensitive potassium (K_ATP_) channels (formed from K_ir_ 6.x and accompanying regulatory sulfonylurea receptor [SUR] subunits). Functionally, all members of this family possess some degree of inward rectification, a characteristic asymmetrical K^+^ conductance whereby K^+^ moves more easily into the cell than out.^25,26^ They tend to be active around E_K_ and thus help set and maintain the resting membrane potential but close in the face of large depolarizations so as not to oppose membrane excitation. For excellent in-depth reviews on K_ir_ channels, see Ashcroft^5^ or Hibino et al.^27^
2. **Two-P K^+^ channels**. These channels have four transmembrane domains and two pore (P) domains per subunit and are therefore referred to as “tandem” or “twin” pore K^+^ channels (K_2P_). The functional channel probably forms as a dimer. Family members include the TWIK (K_2P_1.1), TREK (K_2P_2.1), TASK (K_2P_3.1), THIK (K_2P_13.1), TALK (K2P16.1), and TRESK (K_2P_18.1) channels and constitute “leak” K^+^ conductances.^28^ They are regulated by various stimuli such as pH, O_2_ partial pressure, membrane stretch, temperature, G proteins, fatty acid, and inhalation anesthetics.^29,30^
3. **Voltage-gated K^+^ channels**. These include several important subfamilies: the Shaker, Shab, Shaw, and Shal-related K^+^ channels (K_V_1.x, 2.x, 3.x, and 4.x, respectively); the KCNQ channels (K_V_7.x); and the eag, erg, and elk channels (K_V_10.x, 11.x, and 12.x, respectively). These channels possess six transmembrane domains per subunit with a voltage sensor on the fourth transmembrane segment (S4), which allows them to detect and open in response to membrane depolarization.^31,32^ As such, they tend to play roles in repolarizing membranes in nerve and muscle cells, thus controlling action potential frequency and duration. Four α-subunits come together to form the pore-forming region of the channels and α-subunits usually associate with accessory subunits to form functional channels.
4. **Ca^2+-^activated K^+^ channels**. These share a similar structure to the voltage-gated K^+^ channels but possess an extra transmembrane domain, named S0, involved in regulation by β subunits. The family consists of Ca^2+^-activated Slo (BK) channels (K_Ca_1.x, 4.x, 5.x) and the Ca^2+^-activated SK (K_Ca_2.x) and IK (K_Ca_3.x) channels. These channels are regulated not only by voltage but also by intracellular Ca^2+^; BK_Ca_ channels possess a “calcium bowl” region at the C-terminus, while SK/IK_Ca_ channels are modulated by the calcium binding protein calmodulin.^33,34^

The diversity of K^+^ channels and ability for different subunits within a family to associate to form heteromers often makes it difficult to determine the molecular makeup of channel populations in vivo and thus to assign functional roles. This has been aided to some extent by investigations into human channelopathies, a range of diseases where the genetic disruption of channel subunit activity leads to a distinct phenotype.

## Neuronal K^+^ Channels

Due to the equilibrium potential for K^+^ (mammalian cells ~–85mV), the opening of K^+^ channels generally mediates outward K^+^ currents that act to dampen cellular excitability. Loss of function of several types of K^+^ channel is thus associated with conditions characterized by neuronal hyperexcitability. This includes several forms of epilepsy (**Table 1**), a disorder characterized by abnormal firing of neuronal networks within the brain due to an imbalance between network excitation and network inhibition.

**Table 1.** Neuronal K^+^ Channelopathies.

Protein	Disease	Gene	Effect on Current	Reference
K_V_1.1	Episodic ataxia 1	KCNA1	Loss	36
K_V_3.3	Spinocerebellar ataxia type 13	KCNC3	Loss	42
K_V_4.3	Spinocerebellar ataxia type 19	KCND3	Loss	168
K_V_4.2	Temporal lobe epilepsy	KCND2	Loss	46
K_V_7.1	Jervell and Lange-Nielsen syndrome	KCNQ1/KCNE1	Loss	105, 169–172
K_V_7.2/7.3	Benign familial neonatal convulsions	KCNQ2/3	Loss	52–54
K_V_7.3	Autism spectrum disorders	KCNQ3	Loss	55
K_V_7.4	Autosomal dominant nonsyndromic sensorineural hearing loss DFNA2	KCNQ4	Loss	60, 61
K_V_10.1	Temple-Baraitser syndrome	KCNH1	Gain	173
K_Ca_1.1	Autism spectrum disorders	KCNMA1	Loss	174
K_Ca_1.1	Generalized epilepsy with paroxysmal dyskinesia	KCNMA1	Gain	79
K_Ca_4.1	Epilepsy of infancy with migrating focal seizures	KCNT1	Gain	175
Kv11.1	Epilepsy	KCNH2	Loss	176, 177
K_ir_4.1	SeSAME syndrome (EAST syndrome)	KCNJ10	Loss	93–95
K_ir_6.2	Development delay, epilepsy and neonatal diabetes	KCNJ11	Gain	97
NA	Acquired neuromyotonia	NA		10

EAST, epilepsy, ataxia, sensorineural deafness, and tubulopathy; NA, not applicable; SeSAME, seizures, sensorineural deafness, ataxia, mental retardation, and electrolyte imbalance.

### K_V_1.1-Containing Channels

**Figure 2.**
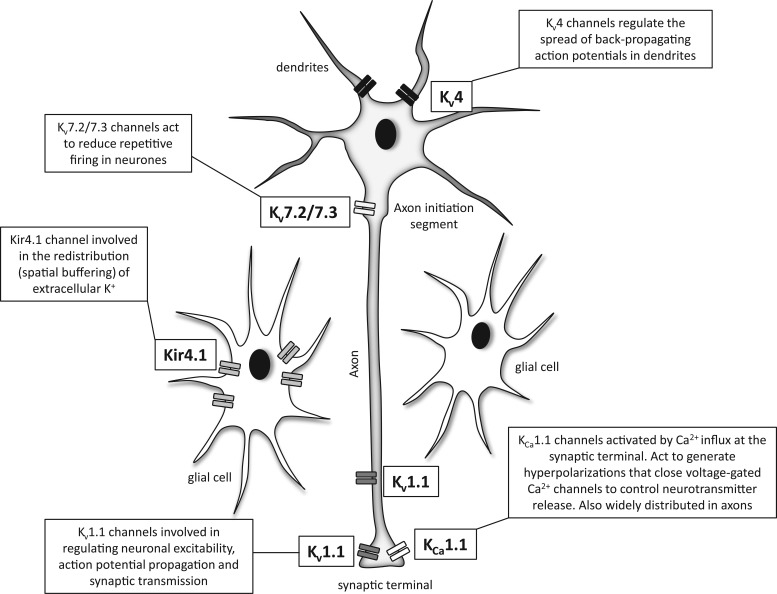
Differential localization of K^+^ channel subtypes in neurons. K_V_1.1-containing channels are expressed in the axon and presynaptic terminal, where they regulate neuronal excitability, action potential propagation, and synaptic transmission. K_V_4.3-containing channels are expressed in dendrites and are involved in regulating the spread of back-propagating action potentials in the dendritic tree. K_V_7.2/7.3 channels, which form the M-current, are expressed in the axon initiation segment and are active at subthreshold membrane potentials where most voltage-gated K^+^ channels are closed. They act to dampen excitability and repetitive firing in neurons. K_Ca_1.1-containing channels are expressed in the presynaptic terminal, where they localize with voltage-gated Ca^2+^ channels. They are activated by the Ca^2+^ influx that occurs in response to action potential–induced terminal depolarization and act to terminate the action potential and generate after-hyperpolarizations that close Ca^2+^ channels and reduce neuronal excitability. K_ir_4.1-containing channels are expressed in glial cells, where they are potentially involved in the redistribution of K^+^. See text for details.

The K_V_1 family includes mammalian homologues of the Shaker K^+^ channels originally cloned in *Drosophila* where mutation of the Shaker (Sh) gene leads to a characteristic trembling of the legs following etherization.^35^ K_V_1.1 is known to associate with other Shaker-related channels (K_V_1.2, K_V_1.4) to form heteromeric channel complexes in various regions of the brain where they control neuronal excitability, action potential propagation, and synaptic transmission (**Fig. 2**).^6^ In humans, a single loss-of-function mutation of K_V_1.1 is associated with episodic ataxia type 1 (EA1), an autosomal dominant neurological condition characterized by continuous involuntary muscle quivering (myokymia) and bouts of severe contractions of head and limb muscles, leading to loss of coordination and balance.^36^ Seizures have been linked to dysfunction of neuronal networks within the hippocampus, a region of the brain located in the medial temporal lobe and often associated with epileptic seizure. Channels containing the K_V_1.1 subunit have been identified in both the axons and synaptic terminals of hippocampal neurons in the rat, and mutations associated with EA1 in humans cause reduced current amplitude and a positive shift in voltage activation of K_V_1.1-containing channels, consistent with reduced channel activity.^37,38^

### K_V_1 Modulators as Drugs

Researchers at Wyeth, now part of Pfizer (New York, NY), have published on novel small-molecule inhibitors of protein-protein interactions that act to modulate K_V_1.1 activity by blocking channel inactivation.^39^ In the hippocampus, K_V_1.1 is coexpressed with accessory K_V_β1 subunits, which convert K_V_1.1 from a slowly inactivating delayed rectifier-type current into a fast inactivating current. This increases neuronal excitability by reducing the sustained hyperpolarizing current that flows through K_V_1.1 and increasing the ability of the neuron to fire repetitively. A novel approach to reducing neuronal excitability is therefore to prevent inactivation of K_V_1.1 channels. A yeast two-hybrid screen identified a number of small-molecule “disinactivators” that most likely interact with sites on the K_V_β1 N-terminus or its receptor site on K_V_1.1 and prevent it binding and inhibiting K_V_1.1 channels.^39^ It is suggested that drugs based on these disinactivators may ultimately be useful for preventing inactivation of K_V_1.1 channels in the brain and thus reducing neuronal hyperexcitability in diseases such as epilepsy. A key advantage of this approach is that, unlike existing anticonvulsants, it would not prevent a neuron from responding to excitatory stimuli but would instead act predominantly to dampen repetitive firing.

There is also therapeutic potential in blocking the activity of functional K_V_1 channels and increasing neuronal excitability. The organic compound 4-aminopyridine (4-AP, fampridine) has been used extensively as a pharmacological tool to study the functional properties of K_V_1 channels for which it is a reasonably selective blocker.^40^ 4-AP (marketed as Ampyra in the United States and Fampyra in Europe) was approved by the Food and Drug Administration (FDA) in 2010 and licensed in the United Kingdom in 2011 for use in the treatment of multiple sclerosis, having been shown to improve walking speed in patients with multiple sclerosis in two clinical trials. 4-AP’s therapeutic effect has not been fully elucidated, but it most likely functions by blocking the prolonged hyperpolarizing currents that flow through K_V_1 channels, shortening the relative refractory period and increasing axonal conduction.

### K_V_3-Containing Channels

Channels in the K_V_3 Shaw subfamily activate rapidly at high-voltage thresholds (~−10 mV) and have very fast deactivation rates. This allows them to open during the peak of the action potential to speed up membrane repolarization and enable repetitive neuronal firing at high frequencies.^41^ K_V_3.3 is expressed throughout the central nervous system, particularly in cerebellar Purkinje neurons. Mutations in K_V_3.3 cause the autosomal dominant neurological disorder SCA13 (spinocerebellar ataxia type 13), which leads to degeneration of the cerebellum and the spinal cord.^42^ The four main disease-associated mutations in K_V_3.3 lead to either reduced channel expression or channels with altered gating properties when expressed in *Xenopus* oocytes.^43^ The reduction in protein levels arises due to a reduction in protein half-life as SCA13 mutations generate unstable proteins that are rapidly degraded. Interestingly, mutant K_V_ 3.3 protein levels could be partially restored by treatment with trimethylamine *N*-oxide, a chemical chaperone that stabilizes the mutant protein and helps folding.^43^ This suggests that identification of small-molecule chaperones may be a novel approach to partially rescuing channel activity.

### K_V_4-Containing Channels

Channels containing K_V_4 Shal subunits mediate the fast-inactivating “A-type” current in dendrites of hippocampal neurons (**Fig. 2**).^44^ These channels are active at subthreshold potentials and are believed to regulate firing frequency and the spread of excitability in the dendritic tree. Action potentials initiated in the axon hillock propagate down the axon but also invade the soma and dendrites (back-propagating action potentials) to inform dendritic synapses that the neuron has fired.^45^ Summation of back-propagating action potentials and excitatory synaptic inputs received by the dendritic tree is believed to be the basis of dendritic signal integration. The activity of dendritic A-type channels limits the spread of back-propagating action potentials and the regulation of K_V_4 expression, localization, and kinetics, thus modulates certain aspects of dendritic signal processing.^44^ Truncation of the K_V_4.2 subunit, resulting in a decrease in dendritic A-current density, has been associated with temporal lobe epilepsy.^46^ The most common form, mesial temporal lobe epilepsy (MTLE), arises in the medial aspect of the temporal lobe where the hippocampus, parahippocampal gyrus, and the amygdala are located. A reduction in dendritic A-current would be expected to lower the firing threshold for action potentials as well as increasing the spread of back-propagating action potentials. Interestingly, seizures have been shown to induce surface recruitment of K_V_4.2 subunits in thalamocortical neurons, which relay sensory information to the cerebral cortex, presumably in a feedback response to reduce excitability.^47^ Pharmacologically, K_V_4 currents are selectively inhibited by several spider toxins that modify gating kinetics^48^ and can be activated by the NeuroSearch (Hellerup, Denmark) compound NS5806.^49^ NS5806 increases current flowing through K_V_4 channels by enhancing peak amplitude and slowing current decay. This latter effect on current inactivation depends on the presence of KChiP2, a cytosolic accessory protein that interacts with the intracellular N-terminus of K_V_4 channels.^49^ Also see the section on cardiac K_V_4 below.

### K_V_7-Containing Channels

Four of the five members of the K_V_7 family are expressed in the nervous system, where they form homomeric and heteromeric K^+^ channels. In many regions of the brain, channels composed of K_V_7.2 and 7.3 subunits underlie the slow voltage-gated “M-current,” so called because the current is inhibited by neurotransmitters acting via G_q_-coupled muscarinic receptors.^50^ These channels localize predominantly to the axon initiation segment in neurons and are open at membrane potentials (from around −60 mV) that are the subthreshold for most voltage-gated K^+^ channels. They do not inactivate and thus generate a steady outward current that stabilizes the membrane in the face of depolarizing currents (reviewed by Brown and Passmore^51^). Since their activation is slow, they tend not to contribute to the repolarization phase of the action potential but act to subdue excitability and repetitive firing in neurons. A number of different mutations of K_V_7.2 and K_V_7.3 have been shown to be associated with idiopathic generalized epilepsy, including benign familial neonatal seizures (BFNS), a disorder characterized by recurrent seizures in newborns.^52–54^ Not all the mutations have been functionally characterized, but most result in reduced amplitude of the M-current, which would be consistent with neuron depolarization and increased burst activity. Seizures in BFNS usually spontaneously stop within the first 15 weeks, although the susceptibility to seizures in later life is increased in BFNS-diagnosed infants (~16% compared with 1%–2% in the general population^52^). It is unclear why seizures cease. One possibility is that the expression of K_V_7 subunits is developmentally regulated, and the neonatal brain is most dependent on the stabilizing effect of the M-current.

Loss of function mutation of K_V_7.3 is also associated with autism spectrum disorders (ASD). In this context, it is probably an M-current formed by the association of K_V_7.3 subunits and K_V_7.5 subunits that is important.^55^ The control these channels exert over neuronal excitability may be important in the generation of synchronous oscillations of networks of neurons, which is believed to be involved in memory formation and storage and, potentially, emotional processing and behavioral monitoring, which are all affected in individuals with ASD.^56–59^ The causal gene for autosomal dominant nonsyndromic sensorineural hearing loss (DFNA2) has been identified as KCNQ4, which encodes the K_V_7.4 protein.^60,61^ This progressive form of hearing loss is thought to result from a decrease in K^+^ efflux from sensory hair cells, potentially leading to damage over time due to K^+^ overload.^62–64^

### K_V_7.2-7.5 Modulators as Drugs

Recently, retigabine (Potiga in the United States and Trobalt in the European Union), a first-in-class K_V_7.2-7.5 opener, has been approved for the use of drug-resistant epilepsy with partial-onset seizures.^65–67^ Retigabine is derived from flupirtine, a drug with longstanding use as a nonopioid analgesic with known relaxant/anticonvulsive properties. Retigabine generates a hyperpolarizing shift in the voltage dependence of channel activation, thus enhancing the stabilizing M-current and limiting neuronal excitability. It shows little selectivity between neuronal K_V_7.2/7.3 channels and other K_V_7.2-7.5 channels and may also affect neurotransmission involving the major inhibitory transmitter gamma-aminobutyric acid (GABA). Retigabine has been shown to increase the concentration of GABA in the brain, by either enhancing GABA synthesis or blocking GABA metabolism, and increases GABA-induced current in rat cortical neurons.^68^ A concerning side effect of retigabine is bladder voiding possibly due to relaxation of bladder smooth muscle (detrusor muscle) or loss of excitability in sympathetic neurons in the bladder.^69^ Other reported side effects include dizziness,^65^ cardiovascular disorders such as prolonging of the QT interval (see later),^70^ and eye pigmentation color change.^70,71^ Interestingly, studies on the retention of patients taking retigabine in the open-label extension study show 60% discontinuation of retigabine treatment at 28 months.^72^ In somewhat different study cohorts, a trial undertaken at University College London predicted a possible near 100% discontinuation of retigabine treatment at 2 years.^73^ ICA-27243, a compound from Icagen (Durham, NC), is reported to be a selective K_V_7.2/7.3 opener that binds to a site in the voltage sensor domain.^74^ In the folded protein, the binding pocket appears to be formed from residues in both the C-terminal end of the S2 domain and the N-terminus of the S3 domain, regions of the channel protein with a high degree of variability between K_V_7 subfamily members.^74^ This highlights the need for improved information regarding the 3D structure of K^+^ channels to identify variable regions that can be targeted by selective modulators.

### K_Ca_1.1-Containing Channels

K_Ca_1.1 (BK) subunits are widely distributed in the axons and at presynaptic terminals of excitatory neurons in the cortex and hippocampus.^75,76^ At synaptic terminals, they are localized in close proximity to voltage-gated Ca^2+^ channels and are activated in response to the Ca^2+^ influx that occurs in response to action potential–induced terminal depolarization.^77,78^ Their activation serves to terminate the action potential and generate after-hyperpolarizations that close Ca^2+^ channels and dampen neuronal excitability. Subunit mutations resulting in loss of channel function would therefore be expected to heighten neuronal excitability consistent with epilepsy. Interestingly, a K_Ca_1.1 mutation discovered by Du et al.^79^ is a gain-of-function mutation associated with generalized epilepsy with paroxysmal dyskinesia (GEPD). This mutation is thought to cause seizures in two possible ways: either K_Ca_1.1-containing channels are expressed in inhibitory neurons, or this gain of K^+^ channel function allows quicker release of Na^+^ channels from inactivation, therefore increasing burst firing of neurons.^80,81^

### K_Ca_1.1 Modulators as Drugs

K_Ca_1.1 channels have proved particularly challenging for drug design (reviewed by Nardi and Olesen^82^). Clinically prescribed drugs such as hydroflumethiazide (Saluron) and chlorothiazide have antihypertensive effects probably because they activate K_Ca_1.1 channels in vascular smooth muscle (see later), but these compounds are essentially diuretics that inhibit Na^+^/Cl^–^ reabsorption from the distal convoluted tubules in the kidneys.^83^ The NeuroSearch activator NS1619 has been used extensively as a pharmacological tool to study K_Ca_1.1 channel function but has poor potency and many off-target effects, most significantly the inhibition of L-type Ca^2+^ channels.^84^ NS11021 is a more selective activator that functions by shifting the voltage activation curve to more negative potentials.^82^ The activation of neuronal K^+^ channels to decrease excitability and neurotransmitter release has been seen as a novel approach for targeting acute ischemic stroke. The activator BMS 204352 (Bristol-Myers Squibb, New York, NY) appeared initially promising and has neuroprotective effects in animal models, significantly reducing cortical infarct volume in a stroke model in spontaneous hypertensive rats.^85^ It reached phase 3 clinical trials for treatment of acute ischemic stroke but failed to show a significant effect over placebo.^85^

### K_ir_4.1-Containing Channels

K_ir_4.1 channel subunits are found primarily on nonneuronal cells within the brain, mostly glial cells within the hippocampus, cortex, thalamus, and brainstem.^86,87^ K_ir_4.1 can form homomeric channels or complex with K_ir_5.1 or K_ir_2.1 to form heteromeric channels. These heteromeric channels show strong inward rectification, unlike the K_ir_4.1 homotetramer. In common with other inward rectifiers, K_ir_4.1 controls the resting membrane potential of astrocytes, and their ability to allow K^+^ to move reasonably freely both into and out of the cell has led to the idea that they help control the microenvironment around neurons by assisting in spatial K^+^ buffering.^88,89^ The restricted extracellular space around neurons means that the repolarization of a single action potential can cause a significant increase in extracellular [K^+^], with high-frequency firing potentially raising extracellular K^+^ by several millimolar.^90^ Due to the high K^+^ permeability of membranes, a prolonged increase in extracellular K^+^ would depolarize neurons and alter excitability. Excess extracellular K^+^ therefore needs to be efficiently siphoned from the immediate vicinity of the neuron, and it is postulated that K_ir_4.1-containing channels allow K^+^ influx into glia at sites of high extracellular K^+^. This K^+^ is then potentially shuttled via a network of gap junction–connected glia and released by efflux through homomeric K_ir_4.1 channels at sites of low extracellular K^+^.^91^ Disruption of this ability to clear K^+^ would have profound effects on neuronal excitability, and there is increasing interest in the role of K_ir_4.1 in epilepsy. Interestingly, glial cells taken during surgery from patients with intractable epilepsy have reduced K_ir_ currents.^92^ Loss-of-function mutation of K_ir_4.1 has also been shown to be associated with seizures, sensorineural deafness, ataxia, mental retardation, and electrolyte imbalance (SeSAME) and epilepsy, ataxia, sensorineural deafness, and tubulopathy (EAST) syndromes.^93–95^ No current modulators are available for these channels.

### K_ir_6.2-Containing Channels

K_ir_6.2 is the pore-forming subunit of the neuronal K_ATP_ channel. These channels respond to fluctuations in intracellular levels of adenine nucleotides and are inhibited by ATP but activated by Mg^2+^-bound nucleotides, particularly MgADP.^96^ This ability to sense intracellular ATP/adenosine diphosphate (ADP) levels ensures that changes in cellular metabolism are translated to changes in membrane K^+^ permeability and thus membrane potential and excitability. A number of K_ir_6.2 gain-in-function mutations give rise to a group of syndromes known as DEND (development delay, epilepsy, and neonatal diabetes).^97^ Twenty percent of DEND patients have neurological disorders such as generalized epilepsy, delay of motor development, and speech and learning disabilities. All characterized mutations share the common feature that they decrease the ability of ATP to close that channel or increase the ability of Mg^2+^ nucleotides to active it. The role of K_ATP_ channels is perhaps best understood in pancreatic β cells, where they are involved in glucose-dependent insulin secretion.^98^ Due to the subunit composition of these pancreatic channels (believed to be K_ir_6.2 in combination with the regulatory subunit SUR1) and the relatively low [ATP]/[ADP] in pancreatic β cells during fasting, pancreatic K_ATP_ channels are constitutively active under basal conditions and help maintain the β-cell resting membrane potential. Elevation of blood glucose results in increased glucose uptake by β cells and its subsequent metabolism, leading to a rise in the intracellular levels of ATP and a fall of ADP. This closes active K_ATP_ channels, resulting in a reduction of K^+^ efflux, depolarization, and activation of L-type voltage-dependent Ca^2+^ channels, which increases Ca^2+^ influx and triggers the Ca^2+^-dependent secretion of insulin. Increased K_ATP_ activity in pancreatic β cells would reduce insulin secretion, explaining the diabetes in these patients, but how hyperactivity of neuronal K_ATP_ channels induces neurological effects is unclear. K_ATP_ channels are expressed predominately in inhibitory GABAergic neurons, with K_ATP_ channel openers causing decreased firing of pyramidal cells in substantia nigra.^99,100^ Drugs that modulate K_ATP_ channel activity are one of the few K^+^ channel clinical success stories and have been extensively reviewed elsewhere.^101^ K_ATP_ channel blockers include the first-generation antidiabetic sulfonylurea tolbutamide, now largely fallen into disuse due to side effects, and the second-generation antidiabetic sulfonylureas glibenclamide, glipizide, gliclazide, glimepiride, and gliquidone. K_ATP_ channel openers include the antihypertensives diazoxide, minoxidil, pinacidil, and the vasodilator nicorandil, which is largely prescribed for the treatment of angina.

## Cardiac K^+^ Channels

**Figure 3.**
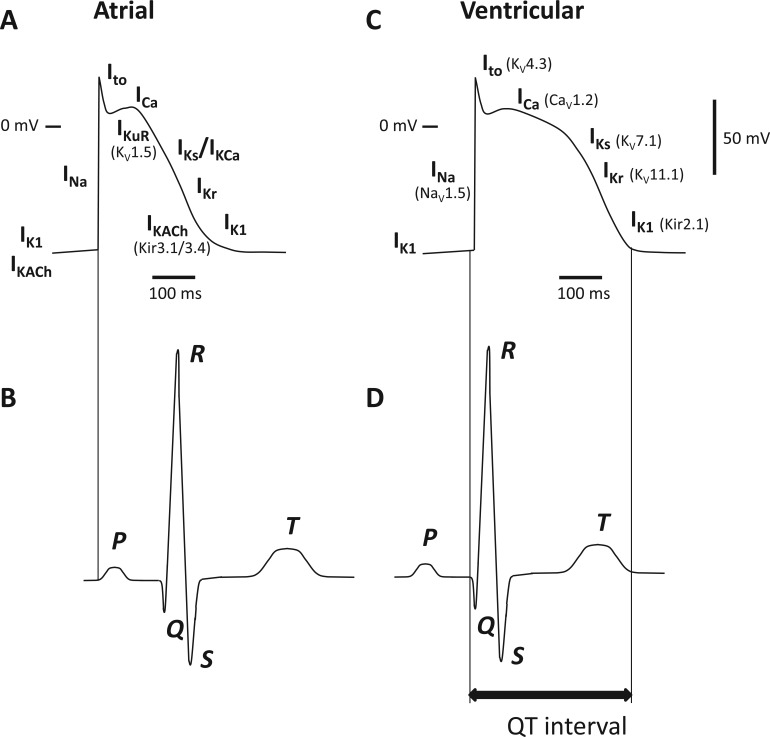
Electrical activity in the atrium and ventricles of the heart. (**A**) Atrial action potential with corresponding underlying currents. Currents similar to ventricle (right) with the exception of atrial-specific currents I_KuR_ (ultrarapid delayed rectifier K^+^ current) mediated by K_V_1.5 channels, I_KACh_ (acetylcholine-regulated K^+^ current) mediated by K_ir_3.1/3.4, and I_KCa_ (calcium-activated K^+^ current) mediated by K_Ca_2.x. (**B**) Relationship between atrial action potential and surface electrocardiogram (ECG). (**C**) Ventricular action potential with corresponding underlying currents. I_Na_ (rapid inward Na^+^ current) mediated by Na_v_1.5; I_to_ (transient outward current) mediated by K_V_4.3; I_Ca_ (inward calcium current) mediated by Ca_v_1.2; I_Ks_ (slow delayed rectifying current) mediated by K_V_7.1; I_Kr_ (rapid delayed rectifying current) mediated by K_V_11.1 and I_K1_ mediated by K_ir_ 2.1. (**D**) Relationship between ventricular action potential and surface ECG.

Disorders of the heart relating to K^+^ channel dysfunction mostly involve disruption of the cardiac action potential and thus the rhythmic contraction of the heart muscle. Alteration of K^+^ channel function results primarily in repolarization disorders such as long QT syndrome (LQTS) and short QT syndrome (SQTS). The synchronized electrical activity of cells within the heart can be recorded on the body surface as the electrocardiogram (ECG). The time elapsed from the beginning of the QRS complex to the end of the T wave on the surface ECG is defined as the QT interval and is largely determined by the length of the ventricular action potential (**Fig. 3**). Prolonged QT interval can produce early after-depolarizations, leading to Torsades de Pointes (TdP), which is the typical arrhythmia associated with LQTS; TdP can in turn generate ventricular fibrillation, a lethal arrhythmia. LQTS results from delayed repolarization of ventricular cells due to a reduction in repolarizing (outward) currents or an increase in depolarizing (inward) currents. It is caused by either loss of function of K^+^ channels or gain of function of Na^+^ or Ca^2+^ channels. SQTS, on the other hand, is caused by gain of function of K^+^ channels.^102^ Mechanisms underlying loss or gain of function vary. Mutations of K^+^ channels have been shown to reduce the number of functional channels at the cell surface by altering trafficking or by affecting the kinetic properties of channel behavior.^103^ LQTS can arise from the disruption of several genes encoding K^+^ subunits (see **Table 2**). These mostly involve mutation of subunits encoding the channels involved in repolarization (I_Ks_, I_Kr_, I_K1_; **Fig. 3**). Repolarization of the atria also involves atrial-specific channels such as K_V_1.5, K_ir_3.1/3.4, and K_Ca_2.2/2.3, which underlie I_KuR_, I_KACh_, and I_SK_ respectively. The limited distribution of these channel subtypes makes them interesting and potentially important targets for the development of novel treatment for atrial fibrillation that do not affect ventricle function.

**Table 2.** Cardiac K^+^ Channelopathies.

Protein	Disease	Gene	Effect on Current	Reference
K_V_7.1	Long QT syndrome 1	KCNQ1	Loss	104, 178
K_V_7.1	Jervell and Lange-Nielsen syndrome type 1	KCNQ1	Loss	105, 172
K_V_11.1	Long QT syndrome 2	KCNH2	Loss	115
MinK protein (minimal potassium subunits)	Long QT syndrome 5	KCNE1	Loss	106
MiRP1 (MinK-related peptide 1)	Long QT syndrome 6	KCNE2	Loss	119
K_ir_2.1	Long QT syndrome 7 (Andersen-Tawil syndrome)	KCNJ2	Loss	179
K_ir_3.4	Long QT syndrome 13	KCNJ5	Loss	140
K_V_11.1	Short QT syndrome 1	KCNH2	Gain	117, 118
K_V_7.1	Short QT syndrome 2	KCNQ1	Gain	108
K_ir_2.1	Short QT syndrome 3	KCNJ2	Gain	180
MiRP2 (MinK-related peptide 2)	Brugada syndrome type 6	KCNE3	Gain	131
MiRP1 (MinK-related peptide 1)	Familial atrial fibrillation type 4	KCNE2	Gain	122
K_V_7.1	Familial atrial fibrillation type 3	KCNQ1	Gain	109
K_V_1.5	Familial atrial fibrillation type 7	KCNA5	Loss	133
K_ir_2.1	Familial atrial fibrillation type 9	KCNJ2	Gain	181
HCN4	Sick sinus syndrome type 2 autosomal dominant	HCN4	Loss	182
K_V_4.3	Brugada syndrome	KCND3	Gain	128
K_V_4.3	Early-onset persistent lone atrial fibrillation	KCND3	Gain	129
MinK protein (minimal potassium subunits)	Early-onset lone atrial fibrillation	KCNE1	Gain	107
K_ir_3.4	Atrial fibrillation	KCNJ5	Loss	183
MiRP2 (MinK-related peptide 2)	Lone atrial fibrillation	KCNE3	Gain	130
MiRP3 (MinK-related peptide 3)	Atrial fibrillation	KCNE4	Gain	184
MiRP4 (MinK-related peptide 4)	Nonfamilial/acquired atrial fibrillation	KCNE5	Gain	185
SUR2A	Paroxysmal Atrial fibrillation	ABCC9	Loss	186

### K_V_7.1-Containing Channels (I_Ks_)

Unlike the other four members of the K_V_7 family, K_V_7.1 is not widely expressed in the CNS but instead is found predominantly in cardiac myocytes and inner ear neurons. In cardiomyocytes, K_V_7.1 (K_V_LQT) combines with the accessory β subunit MinK (KCNE1) to form channels that mediate the slow delayed rectifying K^+^ (I_Ks_) that contributes to the repolarization phase of the cardiac action potential. Loss-of-function mutations of K_V_7.1 lead to long QT syndrome 1 and Jervell and Lange-Nielsen syndrome type 1.^104,105^ These mutations are usually single amino acid missense mutations that cause protein misfolding and early degradation of the channel subunit. In addition to cardiac rhythm defects, patients with Jervell and Lange-Nielsen syndrome have deafness from birth.^105^ Mutation of the accessory protein MinK has been shown to be associated with long QT syndrome 5^106^ and atrial fibrillation,^107^ while gain-of-function mutations of K_V_7.1 are associated with short QT syndrome 2 and familial atrial fibrillation type 3.^108,109^

### K_V_7.1 Modulators

I_Ks_ is a target of interest for the development of antiarrhythmic drugs. Amiodarone, a licensed class III antiarrhythmic, is a nonspecific channel inhibitor that prolongs the cardiac action potential via block of both I_Kr_ and I_Ks_.^110^ K_V_7.1 is insensitive to the anticonvulsant retigabine, which activates other K_V_7 family members, because it lacks a tryptophan residue in the S5 transmembrane domain that is required for retigabine action^111^ (see above). Pharmacologically, K_V_7.1 can be selectively activated by the benzodiazepine L-364,373 (R-L3), which is a partial agonist and increases the amplitude of K_V_7.1 currents as well as slowing the rate of channel activation and deactivation.^112^ Interestingly, most mutant channels associated with long QT syndrome 1 respond similarly to wild-type channels, suggesting that the disease-associated channels would be susceptible to activation.^112^ To our knowledge, the therapeutic benefit of L-364,373 has not been tested. A number of K_V_7.1 selective blockers are known, including L-768,673, HMR1556, and JNJ282 (Johnson & Johnson, New Brunswick, NJ). These have generally shown promising results in animal models, prolonging cardiac action potentials and reducing the incidence of arrhythmias, but have also triggered debate regarding the extension of their usage into humans.^113^ Potential side effects include hearing loss, inappropriate vasoconstriction (see vascular K_V_7.1 below), and the potential to generate TdP and ventricular fibrillation.^113^ We are unaware of any studies investigating selective K_V_7.1 blockade in humans.

### K_V_11.1-Containing Channels (I_Kr_)

K_V_11.1 (hERG1a) subunits associate with subunits produced by an alternative transcript of the KCNH2 gene, termed hERG1b, to form channels that mediate the rapid delayed rectifier current (I_Kr_).^114^ I_Kr_ represents the most important of the repolarizing currents for action potential termination in the ventricles, atria, and cells of the cardiac conduction system. Loss-of-function mutations of K_V_11.1 reduce the amplitude of I_Kr_ and lead to long QT syndrome 2 (LQTS2).^115^ About 300 different K_V_11.1 mutations are linked to LQTS2.^116^ These mutations cause loss of K_V_11.1 channel function by a range of mechanisms, including reducing channel synthesis, suppressing trafficking to the cell membrane, altering channel gating kinetics, or suppressing ion permeation through the channel pore.^116^ Most mutations appear to affect trafficking. Gain-of-function mutations of K_V_11.1 and an increase in repolarizing I_Kr_ current are associated with short QT syndrome 1.^117,118^ When K_V_11.1 is coexpressed with the β subunit MiRP1 in *Xenopus* oocytes, MiRP1 suppresses K_V_11.1 trafficking to the cell surface and accelerates channel deactivation.^119^ In the healthy human heart, MiRP1 is predominantly expressed in the conducting Purkinje fibers, although protein levels have been detected in human ventricles.^120,121^ A loss-of-function MiRP1 mutation is associated with long QT syndrome 6119, while gain-of-function mutations of MiRP1 are associated with familial atrial fibrillation type 4.^122^

### K_V_11.1 Modulators

Limitations in the ability of HTS methods to monitor the complex behavior of the channel has restricted the discovery of activators. Several small-molecule activators of K_V_11.1 have, however, been identified. Type 1 activators such as RPR260243 (originally synthesized by Aventis now part of the Sanofi group) increase K_V_11.1 currents by dramatically slowing channel deactivation (reviewed by Zhou et al.^123^). Type 2 activators such as A935142 (Abbott, Abbott Park, IL), NS1643 (NeuroSearch), ICA-105574 (Icagen), and PD118057 and PD307243 (both Pfizer) primarily impair channel inactivation by binding near the selectivity filter and shifting the voltage dependence of inactivation (reviewed by Zhou et al.^123^). Mallotoxin, a naturally occurring extract from the tree *Mallotus philippinensis*, and KB130015 in contrast accelerate the rate of channel activation. The therapeutic potential of these activators as antiarrhythmics has not been demonstrated clinically. They appear to have off-target effects and may be proarrhythmic and increase the risk of ventricular fibrillation.^124^ Interestingly, some low-affinity K_V_11.1 blocking agents appear to paradoxically restore I_Kr_ by acting as chaperones to transport mutant K_V_11.1 subunits to the membrane.^125^

### K_V_4.3

Channels containing K_V_4 subunits underlie the fast-inactivating “A-type” current I_to_ (**Figure 3**). I_to_ is formed of fast and slow recovering components, I_to1,f_ and I_to1,s_, respectively.^126^ The channel responsible for I_to1,f_ is formed by assembly of K_V_4.2 subunits, K_V_4.3 subunits, or a combination of the two, while the channel responsible for I_to1,s_ is composed of K_V_1.4 subunits. The extent to which alteration of I_to_ can generate arrhythmic activity in the heart has been difficult to ascertain due to a lack of selective blockers or activators. Dynamic clamp of human atrial myocytes, where a current mimicking I_to_ but of opposite polarity was injected into cells, selectively reduced I_to_ and significantly prolonged atrial action potential duration.^127^ In the same study, reduction of I_to_ by dynamic clamp of rabbit atrial myocytes during β-adrenergic stimulation triggered abnormal membrane potential oscillations (after-depolarizations). These could be abolished by dynamic-clamp increases in I_to_ or by application of the β1-antagonist atenolol.^127^ This suggests that changes in I_to_ can potentially provoke arrhythmias. Loss-of-function mutation of the channel subunits underlying I_to_ have not been reported but gain-of-function mutation of K_V_4.3 results in Brugada syndrome^128^ and persistent lone atrial fibrillation.^129^ Mutations of MiRP2, a normally inhibitory β subunit that associates with K_V_4.3, are also linked with lone atrial fibrillation^130^ and Brugada syndrome.^131^ Consistent with this, exposure of ventricular myocytes and ventricular wedge preparations from normal canine heart to NeuroSearch’s K_V_4-selective activator NS5806 mimics the symptoms of Brugada syndrome.^132^

### Atrial K_V_1.5 (I_KuR_)

K_V_1.5 underlies the ultrarapid delayed rectifier K^+^ (I_KuR_) current in the atrium involved in the early stages of atrial repolarization (**Fig. 3**). It represents a potentially important target in treating atrial fibrillation (AF), primarily through the prolongation of the atrial effective refractory period (ERP). The ERP represents the period of time after an action potential has been initiated in which a new action potential cannot generate. During this period, depolarization of cells in the myocardium will not produce significant depolarization in surrounding cells, and the ERP thus acts as a protective mechanism to prevent arrhythmias. Antiarrhythmic agents often act to prolong the ERP, but agents designed to treat AF by prolonging the ERP usually also affect the ventricles, inducing other forms of arrhythmia. Functional currents involved in repolarization of the atrium, but not the ventricles, are thus promising new targets for the development of treatments for AF, and several pharmaceutical companies are currently actively exploring this route. It must be mentioned, while many companies are exploring I_KuR_ inhibitors (see below) for treatment of AF, loss-of-function Kv1.5 mutations have been associated with atrial fibrillation.^133,134^ Loss of Kv1.5 protein has been detected in chronic AF patients; therefore, inhibiting this remaining current may not produce significant effects on ERP in this particular disease state.^135^

### I_KuR_ Modulators

Brivaness (formerly known as Vernakalant or RSD 1235) is a new antiarrhythmic drug recently approved in Europe that inhibits the atrial-specific channels K_V_1.5 and K_ir_3.1/3.4. It has been shown to be effective in terminating acute-onset atrial fibrillation but is relatively nonspecific and can also have some inhibitory effects on I_to_ and I_Kr_ currents.^136^ Bristol-Myers Squibb has a K_V_1.5 inhibitor BMS-919373 in phase 1 trials to study the effects on atrial ERP in patients with a pacemaker (NCT02153437) and in phase 2 trials to assess the effect of BMS-919373 on the time spent in AF (NCT02156076). Pierre Fabre Medicament (Paris, France) has F373280, a novel docosahexaenoic acid derivative and blocker of K_V_1.5 in phase 2 clinical trials for the treatment of persistent AF (NCT01831856). Xention (Cambridge, UK) has the K_V_1.5 blocker XEN-D0101 in a phase 1 proof-of-mechanism electrophysiological study and, in partnership with Servier (Neuilly-sur-Seine, France), the more potent and selective XEN-D0103 in two phase 2 clinical studies.

### Atrial K_ir_3.1/3.4

The acetylcholine-activated K current (I_KACh_) carried by K_ir_3.1/3.4 channels is also a candidate for the development of atrial-specific antiarrhythmics.^137^ The novel compound NTC-801 has been shown to inhibit I_KACh_ with a selectively >1000-fold over other major cardiac currents.^138^ NTC-801 reversed action potential shortening induced by carbachol in isolated guinea pig atrial myocytes but had no effect on ventricular action potential duration. It was also shown to prolong the atrial ERP in a rapid atrial pacing model.^138^ The benzopyrane derivative, NIP-151, is also reported to selectively block I_KACh_ and be capable of atrial-speciﬁc ERP prolongation and stopped AF in two animal models of AF.^139^ In contrast, the same study found that dofetilide, a class III antiarrhythmic used in the treatment of AF, significantly prolonged both atrial and ventricular ERP but had little effect in terminating AF in either model.^139^ While I_KAch_ is predominately thought to be an atrial-specific current, recent research has shown involvement of I_KAch_ in ventricle repolarization, along with a mutation of K_ir_3.4 being associated with LQTS.^140,141^ This may limit the potential of I_KACh_ as a therapeutic target for AF.

### Atrial K_Ca_ 2.x

The calcium-activated K+ current I_KCa_ mediated by KCa2.x has recently been shown to be atrial specific in human hearts142. Blockade of I_KCa_ produces an increase in ERP in sinus rhythm human atrial preparations, whereas in longstanding AF, I_KCa_ blockade has no effect, probably due to the downregulation of K_Ca_2.2/2.3 in longstanding AF.^142^ Interestingly, other studies have found an upregulation in K_Ca_2.x in AF, which leads to speculation that K_Ca_2.x expression is initially increased in AF before downregulation takes place.^143^ Therefore, inhibition of I_KCa_ in recent-onset AF may prove beneficial, as has been shown in paced guinea pig hearts.^144^ To this end, Acesion Pharma (Copenhagen, Denmark) is currently undertaking studies into KCa2.x modulation for the treatment of AF.

## Vascular K^+^ Channels

The primary role of K^+^ channels in the vasculature is to control the resting membrane potential and thus the activity of voltage-gated Ca^2+^ channels, a major Ca^2+^ influx pathway.^145^ In vascular smooth muscle cells, loss or reduction of K^+^ channel activity results in membrane depolarization, increased open probability of voltage-gated Ca^2+^ channels, increased Ca^2+^ influx, and thus contraction and increased vascular tone. An array of different K^+^ channels from all the major families contributes to this role of regulating tone through membrane potential, with channel type and distribution varying markedly with vascular bed and vessel diameter. Mutations in a number of K^+^ channel subunits have been linked with human disease (**Table 3**).

**Table 3.** Vascular K^+^ Channelopathies.

Protein	Disease	Gene	Effect on Current	Reference
K_V_1.5	Pulmonary arterial hypertension	KCNA5	Loss	148
β-1 Subunit K_Ca_1.1	Low prevalence of diastolic hypertension	KCNMB1	Gain	157
K_2P_3.1 (TASK1)	Pulmonary arterial hypertension	KCNK3	Loss	165

### K_V_1.5-Containing Channels

The major voltage-gated K^+^ channels expressed in the vasculature are K_V_1.2, K_V_1.5, K_V_2.1, and K_V_7.4/7.5.^146,147^ Their distribution varies considerably with vascular bed, and there is some controversy over their relative contribution to the regulation of the resting membrane potential. Inhibited gene transcription and/or decreased stability of K_V_1.5 mRNA has been implicated in the reduction of functional K_V_ current in pulmonary artery smooth muscle cells (PASMCs) from patients with primary pulmonary hypertension (PPH).^148,149^ PPH is a relatively rare disease characterized by increased pulmonary vascular resistance and arterial pressure that can ultimately lead to right heart failure. Dependent on the contribution of K_V_1.5 to the resting membrane potential in PASMCs, channel dysfunction might be expected to lead to a membrane depolarization and increased Ca^2+^ influx via activated voltage-gated Ca^2+^ channels. Given the role of K_V_1.5 in the atrial ERP (see above), it seems unlikely that systemically targeting these channels with activators, which would be expected to reduce the ERP, would have significant beneficial effects in these patients over more traditional Ca^2+^ channel-blocking strategies. In this context, it is interesting that targeted introduction of K_V_1.5 into the rat pulmonary circulation by nebulization of an adenovirus carrying the human K_V_1.5 gene reduced pulmonary hypertension.^150^

### K_Ca_1.1 Channels

Increases in intravascular pressure induce a graded depolarization of the smooth muscle cell membrane, which increases the activity of voltage-gated Ca^2+^ channels, raising global Ca^2+^ and initiating contraction.^151^ Although in smooth muscle, the precise mechanism is unclear, Ca^2+^ influx through voltage-gated Ca^2+^ channels also activates ryanodine-sensitive Ca^2+^ release channels (RyRs) located on regions of the sarcoplasmic reticulum in close proximity to the inner side of the plasma membrane.^152^ Localized Ca^2+^ release from single or tightly clustered groups of these channels (subsurface Ca^2+^ sparks) can increase contractility by directly contributing to global Ca^2+^ or by increasing Ca^2+^ entry through membrane depolarization by activating Ca^2+^-activated chloride channels. Ca^2+^ sparks also have a significant negative-feedback effect that acts to limit pressure-induced vasoconstriction.^153^ This is achieved through the activation of plasma membrane K_Ca_1.1 channels. Increases in K_Ca_1.1 channel activity and resultant outward current (spontaneous transient outward currents or STOCs) induce membrane hyperpolarization, which decreases Ca^2+^ entry via voltage-gated Ca^2+^ channels, lowering global Ca^2+^ and exerting a vasorelaxing effect.^152^ A recent study reports that K_Ca_1.1 current density and STOC activity are significantly decreased in vascular smooth muscle cells from patients with hypertension.^154^ While K_Ca_1.1 levels are similar in normotensive and hypertensive individuals, mRNA and protein levels of the β1 subunit KCNMB1 are reduced in arterial tissue from patients with hypertension. This is consistent with previous animal models of hypertension where similar findings have been reported.^155^ Population-based genetic epidemiological studies have also revealed essential hypertension-related genetic variants in the human KCNMA1 gene.^156^ Although no functional deficiency in the K_Ca_1.1 protein was found to explain the association of KCNMA1 genetic variation with an increased risk of systolic severe hypertension, one polymorphism potentially disrupts a binding site for proteins regulating translation and may affect K_Ca_1.1 mRNA levels.^156^ Interestingly, a single-nucleotide substitution in the KCNMB1 gene, leading to a channel gain of function through increased Ca^2+^ sensitivity, is associated with a decreased prevalence of diastolic hypertension.^157^ As mentioned above, the clinically prescribed diuretics hydroflumethiazide (Saluron) and chlorothiazide have off-target antihypertensive effects because they activate K_Ca_1.1 channels in the vascular smooth muscle.^83^ Modulators against these channels would clearly have clinical value, but their broad distribution and history of failed drug design may ultimately make them less attractive targets.^81^

### K_Ca_2.3 and K_Ca_3.1 Channels

A link between genetic variation in the Ca^2+^-activated SK (K_Ca_2.x) and IK (K_Ca_3.x) genes and cardiovascular disease is not well established. These channels are particularly important in the endothelium, where their opening mediates vasorelaxation via the endothelium-derived hyperpolarizing factor (EDHF) pathway (reviewed by Edwards et al.^158^). Here, a rise in endothelial Ca^2+^ induced by the binding of vasodilating mediators to endothelial receptors opens K_Ca_2.3 channels on the endothelial cell surface and K_Ca_3.1 channels located on endothelial projections that protrude through small holes in the internal elastic lamina to make contact with the underlying smooth muscle.^159^ The K^+^ currents that flow out through these open channels induce endothelial hyperpolarization that can spread to subjacent smooth muscle via myoendothelial gap junctions^160^; alternatively, the effluxing K^+^ can activate inwardly rectifying K^+^ (Kir) channels or Na^+^/K^+^-ATPase on smooth muscle cells to induce smooth muscle hyperpolarization and ultimately vasorelaxation.^161^ Selective activation of these channels thus has therapeutic potential for the treatment of conditions such as hypertension, although due to subunit expression in tissues such as the heart (see Atrial K_Ca_2.x section), there are likely to be significant issues with systemic activation. Population analysis has identified several single-nucleotide polymorphisms (SNPs) in both coding and noncoding regions of the K_Ca_2.3 and K_Ca_3.1 genes.^162^ Currently, the only suggestion of genetic linkage to cardiovascular dysfunction is the finding that an intronic SNP in the K_Ca_3.1 gene was significantly less prevalent in a cohort of 313 Japanese patients who had myocardial infarctions than in a control group.^163^

### K_2P_3.1 (TASK1) Channels

A number of members of the K_2P_ family, including TASK-1/2, TREK1/2, TWIK1/2, THIK-1, and TRAAK, have been shown to be present in the vasculature.^164^ These channels are believed to underlie the poorly defined “leak” or background currents and are subject to extensive regulation. Whole-exome sequencing of members of a family with pulmonary arterial hypertension without identifiable mutations in any of the genes usually associated with the disease identified a novel missense variant in KCNK3, which encodes K_2P_3.1 (TASK1).^165^ Five further missense variants in KCNK3 were subsequently identified in unrelated patients with familial pulmonary arterial hypertension and idiopathic pulmonary arterial hypertension.^165^ Functional studies revealed that all these missense mutations resulted in loss of channel function, which could be reversed in most mutants by application of the channel activator, and phospholipase A2 inhibitor, ONO-RS-082. Drugs that pharmacologically inhibit TASK channels include bupivacaine, methanandamide, and Sanofi-Aventis (Paris, France) A293, but as yet there has been little development of selective activators, although a number of patents have been filed focusing on screening and assays using the channel proteins.^101^ The K_2P_ family is a relatively recent discovery and as such represents an area of considerable scope and opportunity for the development of therapeutics.

## Summary

In conclusion, K^+^ channels occupy distinct physiological niches within the human body and have an accessible cell surface location, considerable subunit variability, and often tissue-defined distribution yet have largely evaded successful drug discovery. With the exception of the antidiabetic sulfonylureas and antihypertensives that target K_ATP_ channels, most K^+^ channel modulators in clinical use today are poorly selective and have significant off-target toxicities. One of the reasons for this comparative failure in drug discovery is that these protein complexes are not easy to study. They often gate very quickly, have complex inactivation kinetics, and can be subject to elaborate regulation by voltage and intracellular and extracellular ion concentrations. Many HTS methods rely on indirect measurement of channel activity (ion flux, fluorometric dyes, luminescence) and lack temporal resolution over a physiologically relevant range. The introduction of automated planar-array patch-clamp technology has significantly improved the capacity to track physiological channel activity in response to compound libraries, but potent selective modulators remain elusive. Optimization of lead structures appears difficult, perhaps due to structural restraints imposed by modifiers binding to relatively inaccessible or spatially restricted sites in the channel pore, in regulatory domains, or at the interface with modulatory subunits. Here, in silico modeling and advances in structural biology techniques to crystallize channel proteins within lipid matrices to mimic in vivo open and closed states should generate important data. In addition, crystals of ion channels in complex with modulatory ligands/accessory subunits may reveal key interaction sites and interfaces that can be targeted in drug design. Indeed, targeting interaction interfaces with compounds that either mimic or disrupt the regulatory influence of accessory subunits (see, e.g., the K_V_1.1 “disinactivators”^39^) may ultimately be a more fruitful approach to modulating channel behavior than directly targeting the ion-conducting subunit. It is also worth noting that most disorders associated with loss-of-function mutations in K^+^ channel genes originate not from direct defects in channel activity but from problems with protein folding that lead to early degradation and a reduction in functional channels at the cell surface. An alternative approach may be to identify chaperone agents that stabilize these mutant subunits and allow enhanced trafficking to the membrane. In this context, recent applications have been filed to the FDA and European Medicines Agency (EMA) for a combination therapy for cystic fibrosis using lumacaftor and ivacaftor. Lumacaftor promotes folding of mutated cystic fibrosis transmembrane conductance regulator (CFTR) subunits and increases expression of this Cl^–^ channel at the cell surface. Ivacaftor then acts to increase Cl^–^ conductance in CFTR channels by increasing their open probability.^166^ The applications follow on from two successful phase 3 studies (TRAFFIC and TRANSPORT) that demonstrated significant and sustained improvement in lung function in people with the most common (F508del) form of cystic fibrosis.^167^ The opportunity and need for novel, effective ion channel modulators exists but now need to be matched with innovative design and discovery.
